# Transforming health systems in crisis: innovative infrastructure adaptations for Marburg outbreak response in Biharamulo, Kagera, Tanzania

**DOI:** 10.1136/bmjgh-2025-019661

**Published:** 2026-07-27

**Authors:** Michael Kiremeji, Eliudi Eliakimu, Joseph Hokororo, Naoufel Dridi, Florian Mwebesa, Calvin Sindato, Erasto Sylvanus, James Hellar, Theresia Haule, Erick Richard, Lilian Mreta, Alex Sanga, Erick Stephen Kinyenje, Ruth Ngowi, Radenta Bahegwa, George Mrema, Pascal Muhode, Noel Saitoti, Neema Kamara, Juma Mfinanga, Janeth Masuma, Faraja Msemwa, John Masina, Dick Damas Chamla, Michele Di Marco, Janet Diaz, George S Mgomella, Wangeci Gatei, Ernest Kyungu, Ntuli Kapologwe, Eric Nzeyimana, Said Kilindimo, Leonard Katalambula, Hendry Sawe, Stephen Kibusi, Charles Sagoe-Moses, Grace Magembe

**Affiliations:** 1 Emergency Preparedness and Response, United Republic of Tanzania Ministry of Health, Dodoma, United Republic of Tanzania; 2 Emegency Medicine, Muhimbili University of Health and Allied Sciences, Dar es Salaam, United Republic of Tanzania; 3 Department of Public Health, UDOM, Dodoma, United Republic of Tanzania; 4 Health Quality Assurance Unit, United Republic of Tanzania Ministry of Health, Dodoma, United Republic of Tanzania; 5 MSF, Geneva, Switzerland; 6 National Institute for Medical Research, Dar es Salaam, United Republic of Tanzania; 7 Maweni Regional Referral Hospital, United Republic of Tanzania Ministry of Health, Kigoma, United Republic of Tanzania; 8 Kagera Regional Secretariat, United Republic of Tanzania Ministry of Health, Kagera, United Republic of Tanzania; 9 EPR, Africa CDC, Addis Ababa, Ethiopia; 10 Emergency Medicine, Muhimbili Hospital, Dar es Salaam, United Republic of Tanzania; 11 Tanzania Country Office, WHO, Dodoma, United Republic of Tanzania; 12 Country Office, World Health Organization, Dar es salaam, United Republic of Tanzania; 13 Emergency Preparedness and Response, World Health Organization Regional Office for Africa, Nairobi, Kenya; 14 World Health Organization, Brazzaville, Congo; 15 World Health Organization, Geneve, Switzerland; 16 US Centers for Disease Control and Prevention Research Centers, Dar es salaam, United Republic of Tanzania; 17 Regional Administration and Local Government, United Republic of Tanzania President’s Office, Dodoma, United Republic of Tanzania; 18 ECSA-HC, Arusha, United Republic of Tanzania; 19 Health Department, EAC, Arusha, United Republic of Tanzania; 20 Office of Chief Medical Officer, United Republic of Tanzania Ministry of Health, Dar es Salaam, United Republic of Tanzania

**Keywords:** Health systems, Public Health, Infections, diseases, disorders, injuries, Incidence, Global Health

## Abstract

**Background:**

Marburg virus disease (MVD), a highly fatal haemorrhagic fever with a fatality rate that can reach up to 88%. The outbreak in Biharamulo District in Tanzania in 2025 prompted a rapid transformation of the district hospital into a Marburg treatment unit (MTU). We describe approaches to our innovative solutions and highlight best practices for adapting existing infrastructure in resource-limited and emergency settings.

**Methods:**

We used a mixed-methods approach, integrating observational assessments, facility design analysis, disease containment and clinical data review into the following ‘5S’ framework, that is, systems, security, structure, staff and supplies.

**Results:**

The MTU was systematically organised into green and red treatment zones to prevent cross-contamination. Structural innovations included controlled entry and exit points, a low-risk monitoring area with observation windows and a contactless patient monitoring and medication delivery system. Clinical care strategies focused on implementation of effective systems for triage, early case detection, use of point-of-care (POC) diagnostics, like POC malaria rapid diagnostic test, i-STAT Chem 8, blood gas analysis and bedside ultrasound to guide supportive therapy, including provision of critical care. Deployment of a mobile laboratory to the nearby health facility enabled real-time diagnostics and efficient referral system to improve case management. Staff preparedness and healthcare system’s ability to rapidly expand beyond normal services to meet an increased demand for clinical care during emergencies (surge capacity) were strengthened through infection prevention and control training, basic emergency care training, emergency response drills and psychological support.

**Conclusion:**

The structured response to the Biharamulo MVD outbreak demonstrated the effectiveness of adaptable healthcare infrastructure, evidence-based clinical management and workforce capacity building. These locally-context innovations have highlighted the importance of flexibility, timely intervention and effective collaboration in addressing public health emergencies, while ensuring optimal protection for patients, health staff and the community.

**WHAT IS ALREADY KNOWN ON THIS TOPIC**

- Marburg virus disease is a highly fatal viral haemorrhagic fever (VHF) requiring a rapid, well-coordinated emergency response to prevent widespread transmission.
- Previous VHF outbreaks demonstrated the critical role of timely case isolation, stringent infection prevention and control (IPC) measures and decentralised treatment units in reducing transmission and mortalities.
- Healthcare system resilience, including infrastructure flexibility, rapid diagnostics and staff preparedness, is essential in effectively and efficiently managing VHF outbreaks in resource-limited settings.

**WHAT THIS STUDY ADDS**

- This study provides a detailed account of a rapid transformation of an existing health facility infrastructure into Marburg treatment unit, showcasing an innovative model for adaptive measures for outbreak response.
- It highlights the effectiveness of facility repurposing, including the integration of IPC best practices, structured risk zones, contactless patient monitoring and mobile laboratory diagnostics to enhance case management, prevent nosocomial transmission and outbreak containment.
- The study demonstrates how early case detection, supportive therapy and point-of-care diagnostics improve patient health outcomes.
- Capacity-building initiatives, such as staff IPC training, emergency response drills and psychological support programmes, play a crucial role in sustaining healthcare workforce resilience during outbreaks.

**HOW THIS STUDY MIGHT AFFECT RESEARCH, PRACTICE OR POLICY**

- Findings support the need for pre-established infectious disease treatment units within district hospitals in high-risk areas, ensuring rapid adaptation during outbreaks without disrupting routine healthcare services.
- Policymakers should integrate flexible health infrastructure models that allow for swift facility repurposing in response to emerging infectious diseases.
- Research should focus on scalable, sustainable biosecurity, structural and IPC innovations, including low-cost contactless patient monitoring systems, to enhance safety and efficiency in outbreak settings.

## Introduction

 Marburg virus disease (MVD) is a highly fatal viral haemorrhagic fever (VHF), like Ebola, in the filoviridae family with a fatality rate that can reach as high as 88%.^1 2^ To curb the MVD outbreak, a rapid coordinated emergency response alongside stringent infection prevention and control (IPC) measures is required. MVD outbreaks have occurred sporadically in specific regions since the disease was first identified in 1967 during outbreaks in Marburg, Germany and Yugoslavia, linked to infected monkeys imported from Uganda.^3^ Subsequent outbreaks have been reported in the Democratic Republic of Congo,^4^ Angola,^5^ Uganda^6 7^ and, most recently, Equatorial Guinea in 2023 and Rwanda in 2024. The absence of specific antiviral treatments or approved vaccines further exacerbates its devastating impact.

On 21 March 2023, Tanzania experienced its first MVD outbreak in the Kagera region, located in the northwestern part of the country. The outbreak was triggered by a cluster of illnesses and deaths in Bukoba district, which were confirmed as MVD through laboratory testing. The outbreak lasted for 72 days, ending on 1 June 2023^8 9^ and six patients died. The second outbreak in Tanzania occurred in the same region but in a different district (Biharamulo) on 20 January 2025.^10^

VHF treatment units play a crucial role in responding to these outbreaks by providing a safe environment for optimal patient care and reducing transmission risks through the isolation and treatment of infectious patients in a bio safe healthcare facility.^11^ The need for a designated treatment unit closer to areas of transmission has been emphasised to enhance the quality of care for patients and mitigate possible transmission risks; this has been termed decentralisation. While the advances in clinical care of MVD have significantly evolved and are well documented,^12^ the health system and infrastructure modifications of healthcare facilities during the outbreaks have been rarely addressed.

In response to the MVD outbreak in Tanzania in 2025, a Marburg treatment unit (MTU) was rapidly established within an existing district hospital building constructed in 2018 to enhance outbreak management. All patients accessing healthcare in the hospital were transferred to other nearby facilities to continue with medical care, a critical intervention to prevent human-to-human transmission, which is the predominant form of MVD transmission. The facility was strategically repurposed into a dedicated MTU, with a structured layout designed to optimise patient management and IPC to meet the standards for the highly infectious disease treatment unit (IDTU).^13^ The MTU was organised into multiple treatment zones, effectively separating suspected and confirmed cases while implementing strict protocols to minimise viral transmission, cross contamination and observe biosecurity measures. The design emphasised controlled circulation, clear demarcation between high-risk and low-risk zones, and efficient management of personnel, patients, personal protective equipment (PPE), IPC and medical supplies and waste.

This paper highlights the approaches adapted in the transformation of the Biharamulo district hospital facility into a treatment unit for MVD response, detailing its structural modifications, biosecurity and IPC strategies, and overall impact on the safety of frontline healthcare workers (HCWs), outbreak containment and patient outcomes.

This article aims to document innovative solutions used in transforming an existing hospital into an MTU and to share such best practices for adapting healthcare infrastructure in resource-limited settings for future outbreaks of highly infectious diseases.

### Study design

This is real-time operational research conducted at MTU in Biharamulo, Tanzania, a facility rapidly adapted from an existing hospital structure established in 2018. A comprehensive data collection approach was employed, incorporating observational assessments, facility design analysis and clinical data review.

### Study setting

**Figure 1:**
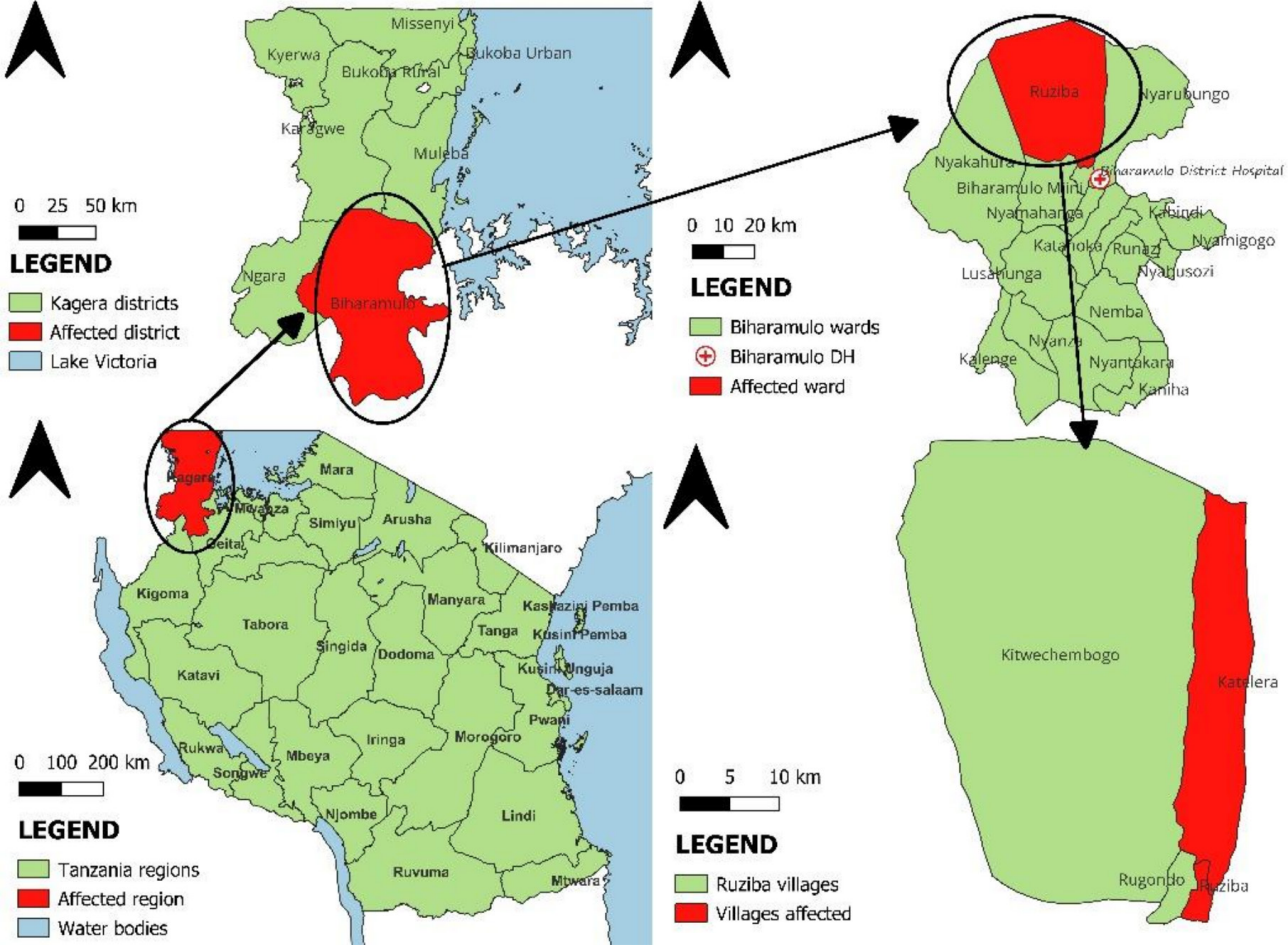
Maps showing MVD affected area, Kagera region. MVD, Marburg virus disease.

Tanzania is a country of 67 million people, with 26 geopolitical regions and 139 districts. Kagera region is one of the 26 regions and is in the north-western part of Tanzania, bordering Uganda in the north, Rwanda and Burundi in the west. Biharamulo district is one of the eight districts of the Kagera region of Tanzania with 457 114 population.^14^ It is bordered to the north by Karagwe district and Muleba district, to the east and south by Geita Region, to the west by Ngara District and to the southwest by the Kigoma Region. Its administrative seat is Biharamulo town. Biharamulo Game Reserve is located within the borders of the district. Biharamulo has 17 wards and the outbreak occurred only in Ruziba ward in which one village was affected. figure 1.

## Material and methods

A mixed-methods study was conducted at the MTU in Biharamulo, Tanzania. The study integrated observational assessments, facility design analysis and clinical data review to evaluate the effectiveness of the MTU in responding to the MVD outbreak. The assessment was structured around the ‘5S’ framework with the following components: security, staff, supplies, structure and system capturing critical aspects of outbreak preparedness and response. The framework describes steps and measures on facility security protocols, workforce capacity, resource availability, infrastructural adaptation and overall system coordination in managing the outbreak. These methodologies facilitated a thorough assessment of the MTU’s effectiveness in outbreak response, infection control measures and overall patient care management.

### Patient and public involvement

Patients and the public were not involved in the design, conduct, reporting or dissemination plans of this research.

### Data collection and management

Data were collected through collaborative efforts involving experts who participated in the outbreak response from the ministry of health (MoH), WHO, WHO-Téchne and Médecins Sans Frontières (MSF). The data were gathered during the 2 months of the MVD response.

Data collection focused on assessing the physical infrastructure modifications, clinical care interventions and staff preparedness measures implemented at the Biharamulo health facility in response to the MVD outbreak management unit. Facility assessments documented the structural modifications, including the designation of isolation units for suspected and confirmed cases, the installation of controlled entry and exit points with decontamination areas and the implementation of biohazard waste disposal systems such as incinerators and dedicated waste collection areas.

## Results

### Innovative structural modifications of the MTU

To effectively manage highly infectious MVD cases, the Biharamulo health facility underwent rapid structural modifications to establish a dedicated isolation unit. Existing hospital wards were repurposed to create distinct zones for suspected and confirmed cases, ensuring strict patient segregation and preventing cross-contamination risks. Comprehensive IPC measures were implemented, including controlled entry and exit points, designated decontamination areas and adherence to rigorous biosecurity standards.

Several innovative adaptations were introduced to enhance safety and operational efficiency within the suspect case building. A low-risk monitoring area was established, allowing HCWs to observe patients through sealed transparent walls, thereby minimising the need for continuous use of full PPE. A unidirectional patient flow system was implemented to guide individuals systematically from suspect to probable case zones and ultimately to confirmed-positive or confirmed-negative areas, significantly reducing the risk of cross-contamination. To address the emotional needs of patients and their families, safe family interaction platforms were set up outside the building, enabling visits at a safe physical distance of at least 2 m. Additionally, a contactless medication delivery system using a secure polyvinyl chloride (PVC) tube and small bucket was employed to transfer medical supplies between low-risk and high-risk zones, ensuring both safety and efficiency in medication administration.

These innovations significantly strengthened IPC measures, optimised patient care workflows and prevented the risk of infection transmission within the facility and to the surrounding community.

### MTU flow control and circulation

**Figure 2:**
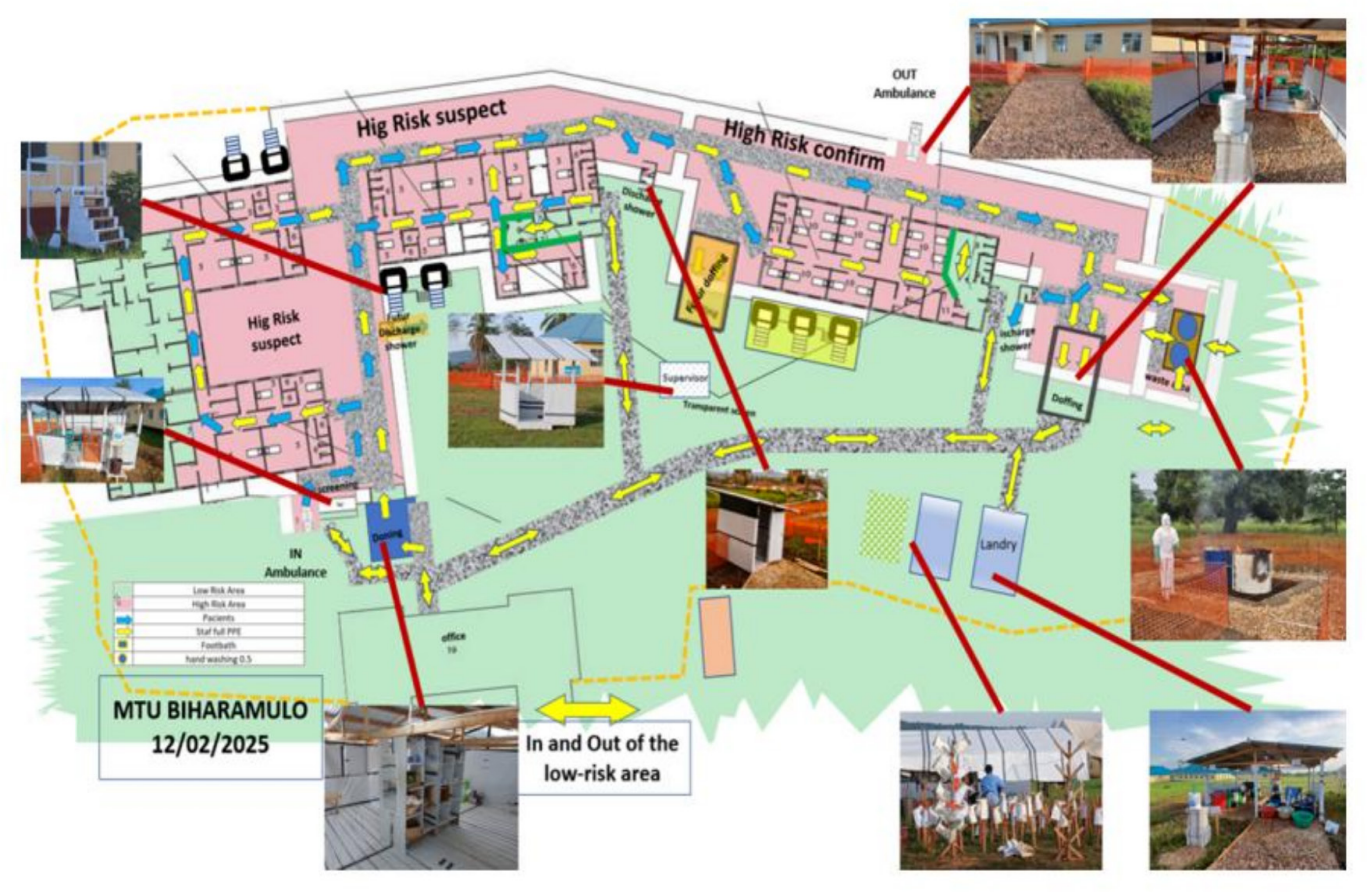
MTU Biharamulo, Tanzania. MTU, Marburg treatment unit; PPE, personal protective equipment.

To enhance optimal biosecurity, IPC and workflow efficiency, the MTU was systematically divided into two distinct zones based on risk profile. This zoning approach was designed in collaboration with IPC specialists and structural engineers to ensure strict infection control measures. Low-risk areas, including administrative offices, staff quarters, pharmacy and storage spaces, were physically separated from high-risk treatment zones through designated unidirectional pathways. Access to high-risk zones from low-risk areas was strictly restricted, preventing any physical interaction between personnel in different risk categories (figure 2).

A suspect building with a 34-bed capacity was designated for patients awaiting diagnostic confirmation, allowing for continuous monitoring before transfer to the confirmed building (if positive test), which accommodated 17 beds. Flow management within the MTU was carefully structured to prevent cross-contamination between staff working in high-risk and low-risk zones. Clearly defined and monitored entry and exit pathways regulated the movement of patients, HCWs, relatives and both clean and contaminated materials. Yellow arrows (figure 2) marked designated pathways, ensuring safe and efficient navigation throughout the facility. Separate exits were allocated for different patient categories and staff groups, further reinforcing safety and operational efficiency.

To support safe movement within the MTU, gravel pathways were laid down throughout the high-risk zone, covering key areas such as donning (PPE application) and doffing (PPE removal) stations, as well as the waste management area. Similarly, pathways in the low-risk zone were clearly marked to direct personnel toward facilities such as the laundry and patient care buildings. This structured approach not only facilitated smooth movement for staff in full PPE but also minimised the transfer of mud, which could compromise the effectiveness of 0.5% chlorine footbaths. Additionally, it reduced the need for excessive daily use of boots, thereby enhancing overall efficiency in infection control measures.

### Innovations in patient care infrastructure

**Figure 3:**
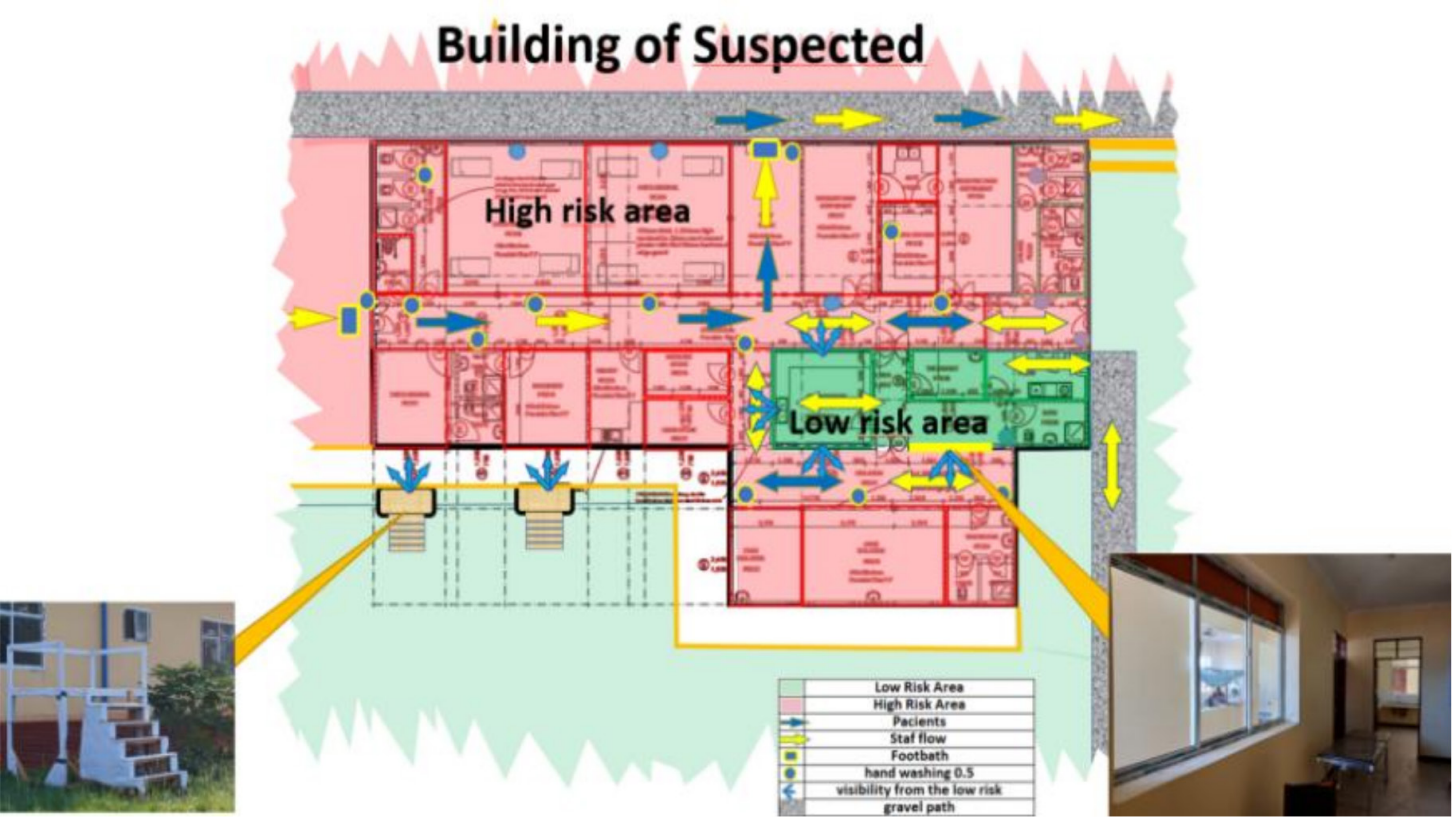
Suspected cases area.

**Figure 4:**
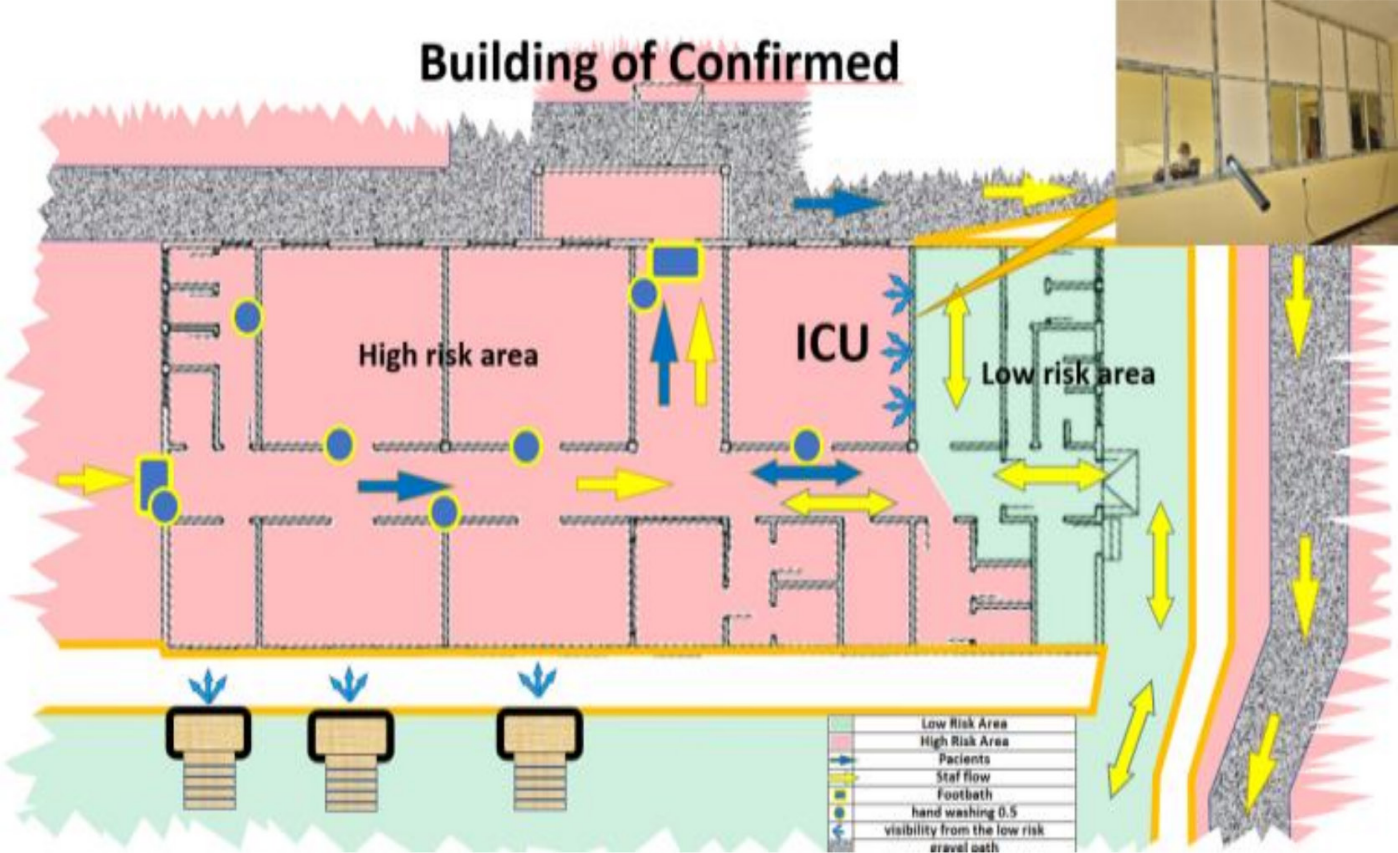
Confirmed cases area. ICU, intensive care unit.

The suspect and confirmed patient buildings, designated for managing patients with MVD, underwent strategic modifications to strengthen IPC measures while optimising patient care and safety. To enhance operational efficiency, a low-risk zone was integrated within the high-risk areas of both the suspect and confirmed buildings (figures 3 and 4).

An aluminium-based material wall (6 m high and 2.40 m wide) with three observation windows was installed in the confirmed cases ward, allowing direct visualisation and continuous monitoring of critically ill patients in the intensive care unit (figure 4). This innovative design enabled HCWs to conduct patient assessments efficiently while reducing the excessive use of PPE, thereby improving both safety and resource management.

Additionally, a PVC tube system was implemented in the suspect area to facilitate the safe, contactless transfer of medications and medical supplies. This system, equipped with a small transport bucket, allowed for secure movement of items from the low risk to high-risk zones, minimising direct contact and further reinforcing IPC protocols (figure 4). These infrastructural innovations significantly enhanced patient care efficiency, optimised resource utilisation and strengthened infection control measures, ensuring a safer environment for both patients and HCWs.

### Clinical care and treatment

The adoption of innovative clinical care and treatment strategies significantly enhanced patient management during the MVD outbreak. A comprehensive screening, triage and early detection system was implemented at the facility entrance, allowing for the prompt identification and isolation of suspected cases. As well, a referral system with dedicated ambulances had a unique entrance to the treatment centre and the MTU could receive patients 24/7. This approach played a critical role in reducing the risk of nosocomial transmission and ensuring timely medical intervention.

Supportive therapy was prioritised, with a strong emphasis on fluid and electrolyte management, blood transfusions for severe haemorrhage and symptomatic treatment to improve patient outcomes. A multidisciplinary team of 20 experienced frontline HCWs from the 2023 MVD response was deployed to the Biharamulo MTU, later joined by 15 newly trained personnel specialising in MVD case management and IPC. This expansion enabled 24-hour operations, strengthening the response capacity and ensuring continuous patient monitoring. A structured approach combining skill diversification, shift-based work schedules with organised team entries into the red zone and ongoing medical education was essential in building clinical and non-clinical staff capacity.

To ensure proactive patient management, clinical meetings were held two times per day and ward rounds were conducted based on patient severity, allowing for real-time treatment adjustments. These strategies contributed to an efficient, adaptive and evidence-based clinical response, optimising patient care while maintaining stringent IPC measures.

PPE usage was reinforced through robust supply chain management and strict adherence to donning and doffing protocols, minimising infection risks for HCWs. A mobile laboratory with VHF diagnostic capabilities was deployed, enabling timely confirmation of suspected cases. Additionally, point-of-care (POC) testing was expanded to facilitate rapid bedside diagnostics, including: malaria rapid diagnostic tests (MRDTs), i-STAT Chem 8 (assessing electrolytes, haemoglobin, urea nitrogen and creatinine), urine dipstick tests, random blood glucose tests (RBG) and bedside ultrasound. The POC testing, and collection of samples for PCR analysis, was performed by trained laboratory personnel. All procedures were conducted in accordance with standard IPC protocols, including the appropriate use of PPE. Waste generated during testing was classified as infectious medical waste and managed in compliance with WHO guidelines for the safe handling and disposal of high-risk biological materials.

These diagnostic tools allowed for the early detection and management of malaria, urinary tract infections, electrolyte imbalances, anaemia and renal insufficiency, expediting treatment initiation and improving patient outcomes.

To enhance critical care capabilities, four high-dependency beds (two in the suspect area and two in the confirmed area) were established to provide standardised care for critically ill patients. As of 28 February 2025, the MTU had admitted 92 suspected cases, with two confirmed MVD cases and 31 malaria cases. No deaths were reported among critically ill suspected cases following the establishment of dedicated critical care beds at the treatment unit, in contrast to the period prior to their setup, during which two deaths occurred. The expansion of POC diagnostics, particularly onsite MRDT and electrolyte testing, enhanced rapid assessment, timely intervention and patient management, ultimately improving clinical outcomes and outbreak preparedness.

### Staff capacity building and preparedness

To ensure the safety of HCWs and the effective management of patients with MVD, immediate capacity-building efforts were implemented. A multidisciplinary response team, comprising epidemiologists, clinicians and public health experts, was established to enhance coordination and decision-making during the outbreak response. Targeted training initiatives were conducted to equip staff with the essential skills needed to manage patients with MVD safely and effectively as follows:

- Basic emergency care training: was provided to 35 MTU staff to strengthen rapid assessment and intervention capabilities.
- Intensive IPC training: was delivered to 58 personnel, covering proper PPE use, safe patient handling and biomedical waste disposal.
- Specialised decontamination training: was conducted for nine hygienist experts to reinforce biosecurity measures.
- Regular emergency response drills: were implemented to enhance staff preparedness for potential case surges.
- Psychological support and stress management training: was integrated into the programme to promote mental well-being and resilience among HCWs.

### Healthcare system readiness and gap assessment

Beyond the MTU, case management and IPC readiness assessments were conducted across 42 health facilities using WHO assessment tools to identify gaps in outbreak preparedness. Key challenges identified included:

- Lack of patient screening protocols for individuals entering health facilities.
- Absence of designated holding rooms for suspected cases.
- Inadequate handwashing facilities, compromising IPC compliance.

To address these gaps, 102 healthcare professionals—including clinicians, nurses, laboratory scientists, health assistants, social welfare technicians and IPC experts—were trained. This initiative aimed to strengthen outbreak response capacity and IPC measures across the healthcare system, ensuring a more coordinated and effective approach to future public health emergencies**,** highlighting the importance of screening and isolation capacity throughout the health system.

### Infectious disease unit proposed plan as Kagera MVD case study

**Figure 5:**
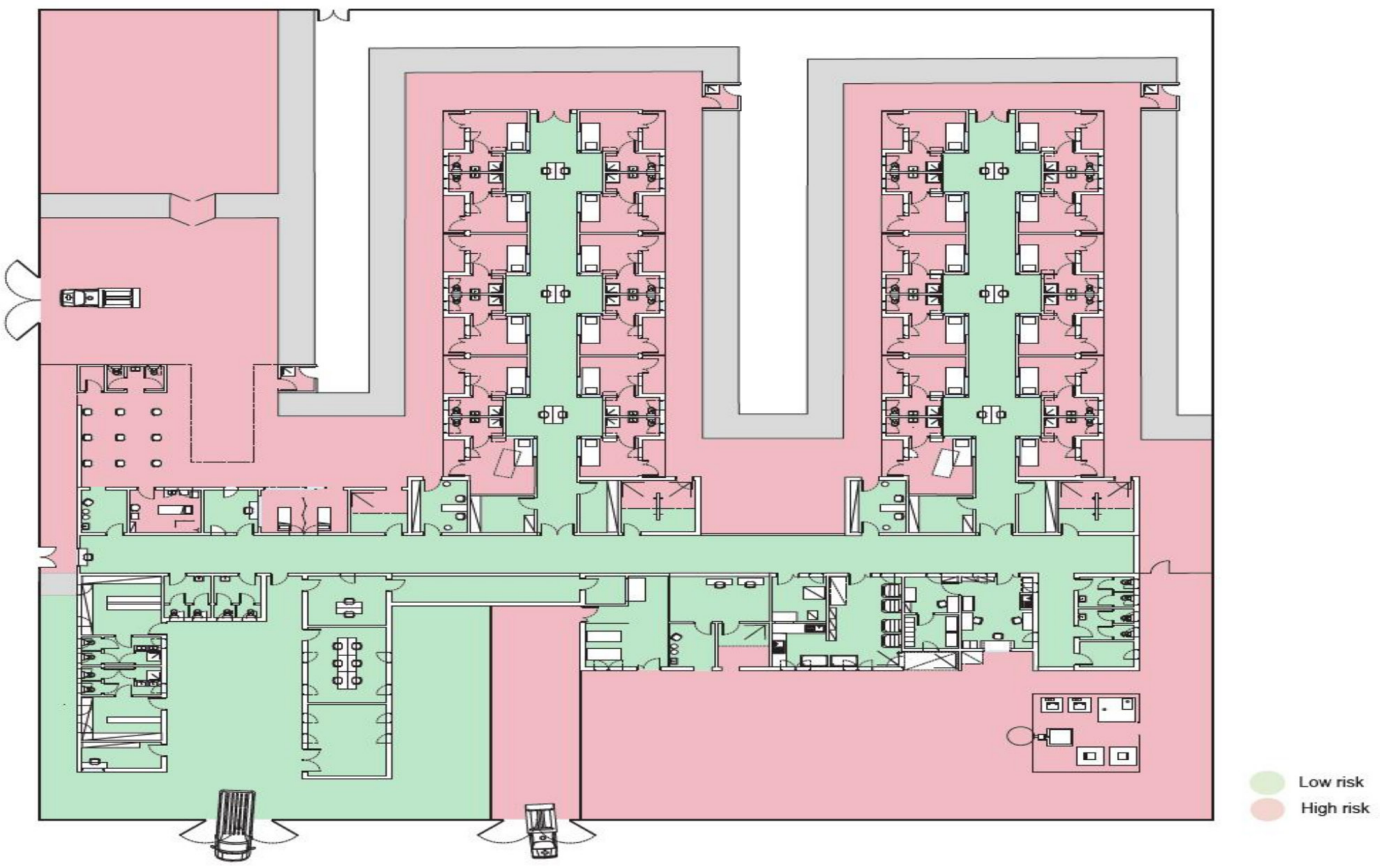
Infectious disease unit Tanzania future plan.

The MVD outbreak in Kagera highlighted the urgent need to decentralise IDTUs. This need became evident from the experiences of the 2023 and 2025 MVD outbreaks. During the 2023 outbreak the Bujunangoma district hospital was repurposed as an MTU. Following the conclusion of these outbreaks, the MTU was decommissioned and the hospital resumed its routine operations. Similarly, during the current outbreak, Biharamulo district hospital has been repurposed to serve as an MTU. Drawing from these experiences, the MoH case management and IPC team, in close collaboration with WHO and WHO-Techne has proposed a model for establishing dedicated IDTUs within district hospitals in all high-risk districts (figure 5). This approach ensures proximity to outbreak epicentres, enabling rapid case isolation, timely treatment and improved response times. During non-outbreak periods, these units will function as part of routine hospital operations, while during outbreaks, they will be swiftly repurposed to manage infectious diseases.

## Discussion

The response to MVD outbreak in Biharamulo District exemplifies a well-coordinated approach integrating innovative patient care strategies, biosecurity and IPC measures, and healthcare workforce preparedness. This approach is similar to the past initiatives including Ebola virus disease (EVD) in West Africa (2014–2016)^15 16^ and the Democratic Republic of Congo (2018–2020),^17 18^ where rapid infrastructure adaptation and IPC enhancements were crucial in outbreak containment. A key innovation in the Biharamulo response was the rapid transformation of the health facility into an MTU. This strategy aligns with lessons learnt from the 2014 EVD outbreak, where flexible infrastructure adaptation was essential for effective patient segregation and disease containment.^19–21^ The repurposing of existing hospital wards into specialised zones for suspected and confirmed cases minimised cross-contamination and streamlined patient management.

The design and organisation of MTU in Biharamulo integrated advanced IPC principles for delivery of optimised clinical care. The establishment of distinct risk zones, including designated pathways and exits for different personnel and patient categories, provides additional evidence on the best practices observed in the West African EVD outbreak, where structured treatment centres reduced nosocomial transmission.^22^ The implementation of a low-risk monitoring area within the suspect building and the use of contactless medication delivery via PVC tubes were novel adaptations enhancing operational safety and efficiency. The installation of observation windows allowed continuous patient monitoring while conserving PPE, a strategy successfully applied in previous VHF outbreaks to optimise resource use; which is also a key action for minimising health systems effects to climate towards ‘achieving net zero emissions in the health sector’.^23–26^

The implementation of innovative clinical care and treatment strategies during MVD outbreak significantly enhanced patient outcomes and strengthened overall outbreak response capacity. Early case detection, efficient referral system and triage at the facility entrance proved to be a critical intervention in reducing nosocomial transmission, a common challenge in VHF outbreaks.^12 27^ The integration of supportive therapy, particularly fluid and electrolyte management, blood transfusions, symptomatic treatment and care to the critically ill patients aligned with best practices in the management of severe viral infections.^28^ By reinforcing PPE supply chains and enforcing strict donning and doffing protocols, the risk of healthcare-associated infections was minimised, ensuring the safety of frontline workers at Biharamulo MTU.

The experience from past MVD outbreaks in Kagera has demonstrated the need for decentralised IDTUs in district hospitals, particularly in high-risk areas. Similar lessons have been observed in previous outbreaks, such as the 2005 MVD outbreak in Angola, where delayed case isolation contributed to high mortality.^29^ During the 2014–2016 EVD outbreak in West Africa, studies showed that decentralising treatment centres improved patient outcomes and reduced transmission rates.^30^ Repurposing hospitals like Bujunangoma and Bihalamulo during outbreaks has proven efficient and effective but also highlighted challenges. To address this, the case management and IPC teams propose a structured model where IDTUs function as routine hospital units during non-outbreak periods and are swiftly repurposed for infectious disease management when needed. This approach ensures rapid response, HCWs safety and community, improves patient outcomes and strengthens healthcare system resilience, ultimately enhancing outbreak preparedness and containment.

A key strength of the response was the deployment of a mobile laboratory capable of diagnosing VHFs in real-time. This innovation, coupled with enhanced POC investigations, improved diagnostic accuracy and enabled timely clinical decision-making. The use of bedside investigations such as POC MRDT, i-STAT Chem 8, urine dipstick, RBG and bedside ultrasound provided rapid diagnostic capabilities, reducing delays in treatment initiation.

The structured approach to patient management, including two times per day clinical meetings and ward rounds based on patient severity, facilitated dynamic treatment adjustments and continuous monitoring, ultimately optimising patient care. These practices align with established supportive care and IPC guidelines, reinforcing the importance of evidence-based decision-making in emergency response settings.^19 27 31^

Staff training and preparedness played a crucial role in the outbreak response. The rapid availability and deployment of an experienced workforce from previous outbreak and establishment of a multidisciplinary response team fostered improved coordination and decision-making. Collaboration between various actors including MOH, WHO and MSF ensured quality in all aspects. The training of the MTU staff in basic emergency care, along with intensive IPC training and specialised decontamination training for nine hygienist experts, contributed to a robust IPC measures implementation. Conducting emergency response drills enhanced HCWs’ readiness to manage patient surges effectively. Psychological support and stress management training were also integrated, recognising the mental health impact of outbreak response on frontline workers.

Beyond the MTU, outbreak preparedness efforts were expanded to 42 of Biharamulo health facilities, where IPC assessments identified gaps and informed targeted training initiatives. The training of the healthcare professionals, including clinicians and nurses, strengthened outbreak response capacity across the healthcare system. This decentralised approach is aligned with global health security recommendations, emphasising the need for system-wide resilience in public health emergency preparedness. Overall, the structured and multidisciplinary response to the MVD outbreak underscores the importance of integrating clinical best practices, rapid diagnostics and comprehensive staff training in outbreak management.

The response to the MVD outbreak in Biharamulo revealed several critical lessons to inform future preparedness efforts. Rapid adaptation of the healthcare system, including the swift transformation of Biharamulo District Hospital into a MTU, proved essential for efficient outbreak response, consistent with findings from prior outbreaks where health systems’ flexibility was linked to improved outcomes.^8^ Structured IPC measures significantly improved disease containment, aligning with evidence from Ebola and COVID-19 responses that underscore IPC’s role in minimising nosocomial transmission.^32^ The availability of trained and committed staff was crucial for both patient outcomes and preventing transmission, supporting literature that highlights workforce capacity as a cornerstone of epidemic control.^21 33^ The deployment of a well-prepared emergency medical team ensured continuity and quality of care, reflecting WHO recommendations on the importance of rapid response teams in outbreak settings.^34^ Additionally, resource-efficient patient monitoring strategies enhanced care delivery even in resource-limited settings, echoing studies advocating for pragmatic approaches to clinical management in low-resource environments.^35^ Comprehensive staff training contributed to healthcare system resilience, reinforcing calls for continuous capacity-building as a fundamental element of epidemic preparedness.^36^ Collectively, these experiences emphasise the need for sustained investments in outbreak preparedness, flexible infrastructure and health workforce capacity-building to strengthen health system resilience and mitigate the impact of future epidemics.

## Conclusion

The MVD outbreak response in Biharamulo District demonstrated the effectiveness of a multidisciplinary approach that integrated innovative patient care, stringent IPC measures and comprehensive healthcare workforce preparedness. Rapid infrastructure adaptation, structured risk zoning and novel strategies like contactless medication transmission enhanced patient safety and operational efficiency. The deployment of mobile laboratories and POC diagnostics ensured timely clinical decision-making, while extensive staff training and psychological support reinforced frontline resilience. Expanding IPC interventions across multiple health facilities further strengthened system-wide preparedness. This experience underscores the importance of adaptable infrastructure, evidence-based clinical management and workforce capacity building in outbreak response, providing a valuable framework for future public health emergency preparedness in resource-limited settings.

## Data Availability

Data sharing not applicable as no datasets generated and/or analysed for this study.

## References

[1] World Health Organization Marburg virus disease. https://www.who.int/news-room/fact-sheets/detail/marburg-virus-disease.

[2] Brauburger K, Hume AJ, Mühlberger E (2012). Forty-five years of Marburg virus research. Viruses.

[3] Siegert R, Shu HL, Slenczka W (1946). Isolation and identification of the “Marburg virus. Dtsch Med Wochenschr.

[4] Bausch DG, Borchert M, Grein T (2003). Risk factors for Marburg hemorrhagic fever, Democratic Republic of the Congo. Emerg Infect Dis.

[5] Towner JS, Khristova ML, Sealy TK (2006). Marburgvirus genomics and association with a large hemorrhagic fever outbreak in Angola. J Virol.

[6] Adjemian J, Farnon EC, Tschioko F (2011). Outbreak of Marburg hemorrhagic fever among miners in Kamwenge and Ibanda districts, Uganda, 2007. J Infect Dis.

[7] Ezie KN, Takoutsing BD, Modeste D (2024). Marburg virus outbreak in Equatorial Guinea: need for speed. Ann Glob Health.

[8] Kinyenje E, Hokororo J, Ngowi R (2024). Infection prevention and control of highly infectious pathogens in resource-limited countries: an experience from Marburg viral disease outbreak in Kagera Region - Tanzania. BMC Infect Dis.

[9] Patel VA, Verma A, Verma M (2025). Marburg virus disease in Tanzania: a review. Clin Infect Pract.

[10] United Republic of Tanzania Ministry of Health Marburg virus disease situation report. https://www.moh.go.tz/storage/app/uploads/public/679/d11/e98/679d11e98e421942331743.pdf.

[11] Fontana L, Pagano F, De Filippi F (2024). Evolution of Ebola and Marburg treatment centers design: a review of the last ten years of outbreaks in Africa. Architecture.

[12] Kreuels B, Wichmann D, Emmerich P (2014). A case of severe Ebola virus infection complicated by gram-negative septicemia. N Engl J Med.

[13] The Lancet The best science for better lives. https://www.thelancet.com/.

[14] National Bureau of Statistics, Tanzania 2022 population and housing census: initial results. https://www.nbs.go.tz/uploads/statistics/documents/sw-1720088450-2022%20PHC%20Initial%20Results%20-%20English.pdf.

[15] Spengler JR, Ervin ED, Towner JS (2016). Perspectives on West Africa Ebola virus disease outbreak, 2013–2016. Emerg Infect Dis.

[16] Kyobe Bosa H, Kamara N, Aragaw M (2024). The West Africa Ebola virus disease outbreak: 10 years on. Lancet Glob Health.

[17] Ilunga Kalenga O, Moeti M, Sparrow A (2019). The ongoing Ebola epidemic in the Democratic Republic of Congo, 2018–2019. N Engl J Med.

[18] Fontana L, Ondo Avomo CO, Ngomo Mikue LE (2024). Case series of patients with Marburg virus disease, Equatorial Guinea, 2023. N Engl J Med.

[19] Wendelboe AM, McCumber M, Erb-Alvarez J (2018). Managing emerging transnational public health security threats: lessons learned from the 2014 West African Ebola outbreak. Global Health.

[20] Coltart CEM, Lindsey B, Ghinai I (2017). The Ebola outbreak, 2013–2016: old lessons for new epidemics. Phil Trans R Soc B.

[21] Dembek Z, Hadeed S, Tigabu B (2024). Ebola virus disease outbreaks: lessons learned from past and facing future challenges. Mil Med.

[22] Chowell G, Viboud C (2015). Controlling Ebola: key role of Ebola treatment centres. Lancet Infect Dis.

[23] Royal College of Surgeons Two years on: tackling the environmental impact of PPE. https://www.rcseng.ac.uk/news-and-events/blog/tackling-environmental-impact-ppe/.

[24] Kociubinska J (2022). Human health, climate change and PPE use during the COVID-19 pandemic. Br Dent J.

[25] Cubas ALV, Moecke EHS, Provin AP (2023). The impacts of plastic waste from personal protective equipment used during the COVID-19 pandemic. Polymers (Basel).

[26] Lyu L, Bagchi M, Markoglou N (2024). Innovations and development of sustainable personal protective equipment: a path to a greener future. *Environ Syst Res*.

[27] Lyon GM, Mehta AK, Varkey JB (2014). Clinical care of two patients with Ebola virus disease in the United States. N Engl J Med.

[28] World Health Organization Therapeutics for Ebola virus disease. https://www.who.int/publications/i/item/9789240055742.

[29] Ligon BL (2005). Outbreak of Marburg hemorrhagic fever in Angola: a review of the history of the disease and its biological aspects. Semin Pediatr Infect Dis.

[30] Médecins Sans Frontières (2024). Ebola: we are better prepared to respond to an epidemic. https://epicentre.msf.org/en/our-achievements/ebola-we-are-better-prepared-respond-epidemic.

[31] Leligdowicz A, Fischer WA, Uyeki TM (2016). Ebola virus disease and critical illness. Crit Care.

[32] Theocharopoulos G, Danis K, Greig J (2017). Ebola management centre proximity associated with reduced delays of healthcare of Ebola Virus Disease (EVD) patients, Tonkolili, Sierra Leone, 2014-15. PLoS ONE.

[33] Raven J, Wurie H, Witter S (2018). Health workers’ experiences of coping with the Ebola epidemic in Sierra Leone’s health system: a qualitative study. BMC Health Serv Res.

[34] World Health Organization Regional Office for the Eastern Mediterranean Country health emergency preparedness and International Health Regulations. http://www.emro.who.int/international-health-regulations/overview/overview.html.

[35] Serrano LP, Maita KC, Avila FR (2023). Benefits and challenges of remote patient monitoring as perceived by health care practitioners: a systematic review. Perm J.

[36] Kruk ME, Ling EJ, Bitton A (2017). Building resilient health systems: a proposal for a resilience index. BMJ.

